# Broadly-Neutralizing Antibodies Against Emerging SARS-CoV-2 Variants

**DOI:** 10.3389/fimmu.2021.752003

**Published:** 2021-09-27

**Authors:** Lok Bahadur Shrestha, Nicodemus Tedla, Rowena A. Bull

**Affiliations:** ^1^ School of Medical Sciences, Faculty of Medicine, Sydney, NSW, Australia; ^2^ The Kirby Institute, Faculty of Medicine, Sydney, NSW, Australia

**Keywords:** SARS-COV-2 variants, broadly neutralising antibodies, delta variant, monoclonal antibody, CDR

## Abstract

The emergence of severe acute respiratory syndrome coronavirus 2 (SARS-CoV-2) variants have become a major concern in the containment of current pandemic. The variants, including B.1.1.7 (Alpha), B.1.351 (Beta), P1 (Gamma) and B.1.617.2 (Delta) have shown reduced sensitivity to monoclonal antibodies, plasma and/or sera obtained from convalescent patients and vaccinated individuals. Development of potent therapeutic monoclonal antibodies (mAbs) with broad neutralizing breadth have become a priority for alleviating the devastating effects of this pandemic. Here, we review some of the most promising broadly neutralizing antibodies obtained from plasma of patients that recovered from early variants of SARS-CoV-2 that may be effective against emerging new variants of the virus. This review summarizes several mAbs, that have been discovered to cross-neutralize across Sarbecoviruses and SARS-CoV-2 escape mutants. Understanding the characteristics that confer this broad and cross-neutralization functions of these mAbs would inform on the development of therapeutic antibodies and guide the discovery of second-generation vaccines.

## Introduction

**Figure 1:**
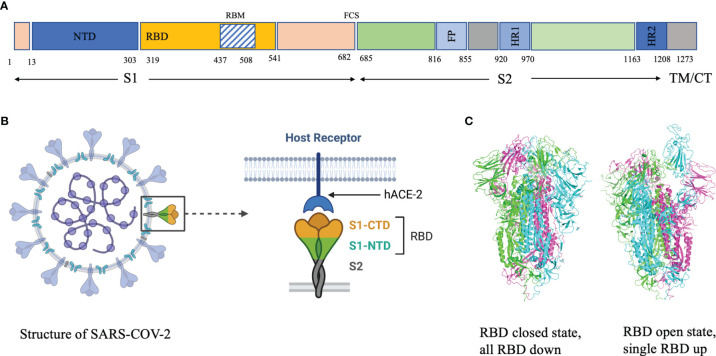
**(A)** Domain architecture of SARS-COV-2 spike protein. **(B)** Structure of SARS-COV-2 virus and interaction of the receptor binding domain with human angiotensin converting enzyme 2 (hACE-2). **(C)** Trimeric spike protein showing RDB in closed state (down conformation, left) and open state (up conformation, right). Each color represents a monomer. RBD, receptor binding domain; CTD, C-terminal domain; NTD, N-terminal domain, hACE-2, human angiotensin converting enzyme-2; S1, spike protein 1; S2, spike protein 2; CT, Cytoplasmic tail; TM, transmembrane domain; FCS, Furin cleavage site, the numbers refer to amino acid residues. Figure created using Biorender, Pymol and Microsoft PowerPoint.

Analysis of the breadth and potency of neutralizing antibodies (nAbs) elicited by severe acute respiratory syndrome coronavirus 2 (SARS-CoV-2) infection is vital for understanding the quality of immune protection and unraveling optimal targets for therapeutic or vaccine design (1). SARS-CoV-2 nAbs most commonly target the Spike glycoprotein, which interacts with the angiotensin-converting enzyme 2 (ACE2) receptor on the host-cell surface to facilitate virus entry (**Figure 1**). The Spike glycoprotein oligomerises three copies of both the S1 and S2 subunits. The S1 subunit mediates binding to ACE2 and the S2 subunit arbitrate fusion with host-cell membrane. Each S1 subunit can be further divided into an N-terminal domain (NTD) and a receptor-binding domain (RBD). RBD contains a core and a receptor-binding motif (RBM); the RBM mediates contact with ACE2 (2, 3). RBDs can adopt either ‘down’ or ‘up’ conformations, but interaction with ACE2 is usually plausible only in an ‘up’ conformation (4–6). The majority of the more potent neutralizing monoclonal antibodies (mAbs) characterized to date bind the RBD of the viral spike protein (5–11), though some mAbs against the NTD have also been described (12, 13).

**Figure 2:**
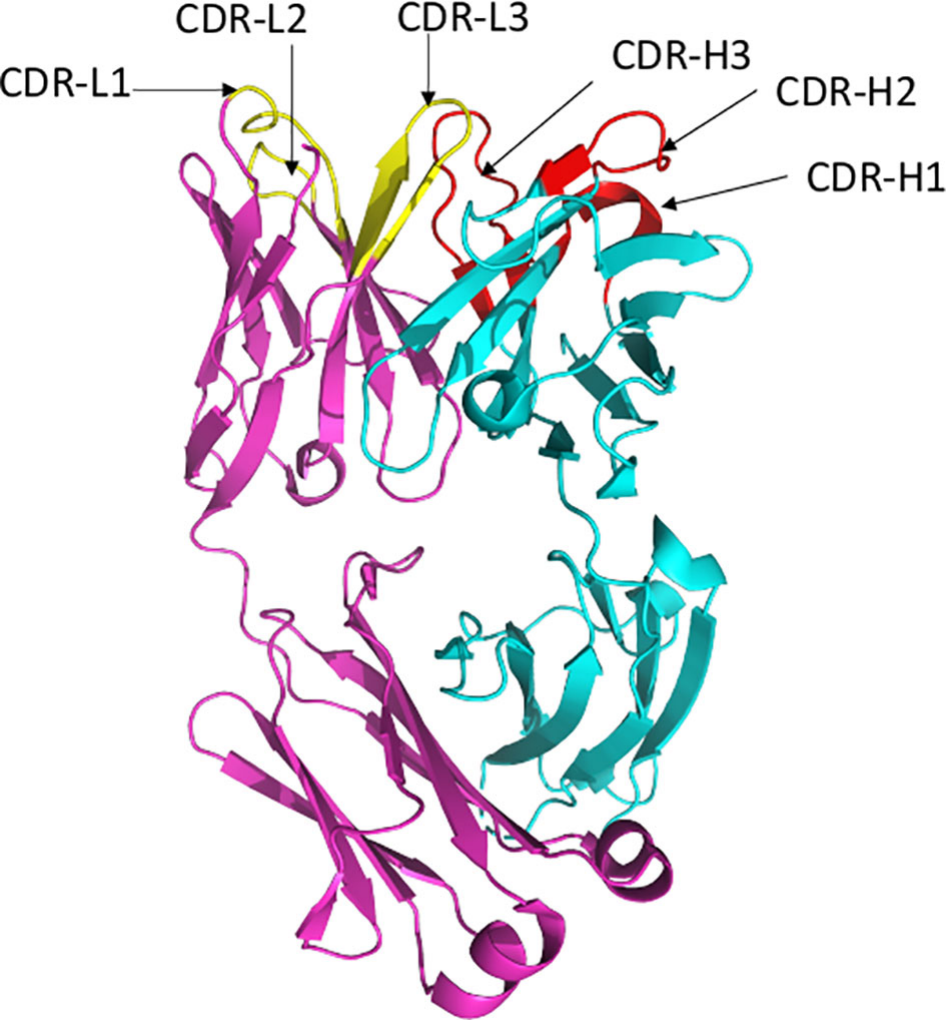
Ribbon representation of Fab region of a human antibody. The heavy chains are shown in cyan, while light chains are shown in pink. Complementarity determining regions (CDR) of heavy chain (CDRH1, CDR-H2, CDR-H3) are shown in red, CDR of light chain (CDR-L1, CDR-L2, CDR-L3) are shown in yellow. Figure drawn by using Pymol; residues obtained from PDB (www.rcsb.com) using following PDB ID 7CR5.

Antibodies bind their epitopes using diversified loops, termed complementarity-determining regions (CDRs) rearranged within the variable regions of heavy (VH) and light (VL) chains of the immunoglobulin molecules (**Figure 2**). CDRs 1 and 2 are encoded by germline V genes, while CDR3s in both VH and VL regions are the product of gene recombination. The J genes are involved in the production and diversity of light and heavy chains by influencing the composition of the variable regions (VDJ and VJ). Compared to the other CDRs, the variation in possible length and biochemical properties of the heavy-chain complementarity-determining region 3 (CDR3-H3) contribute to enhanced diversity in antigen recognition (14, 15). Genetic characterization of SARS-CoV-2 nAbs described to date reveal enrichment of specific antibody-variable genes, including VH3-53, VH3-66, VH1-2, VH1-69, VH1-58, and VH3-30 (16–17); VH3-53 and VH3-66 being the most commonly reported class of mAbs (17).

**Figure 3:**
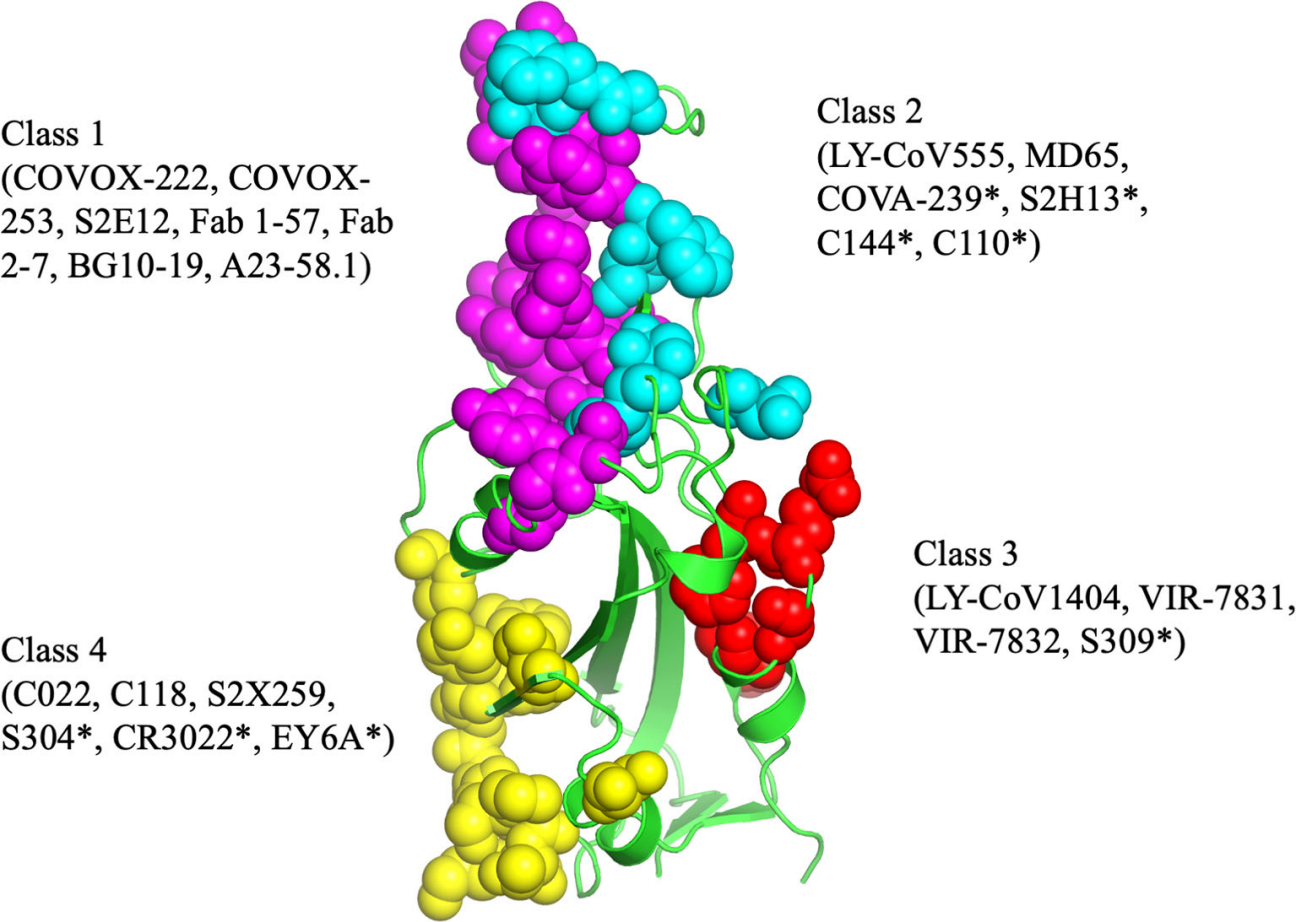
The antibody binding epitopes in the RBD of SARS-COV-2 as classified by Barnes et al. Class 1 antibodies are represented as spheres in pink color; class 2 as cyan; class 3 as red and class 4 as yellow. Figure drawn by using Pymol; residues obtained from PDB (www.rcsb.com) using following PDB ID (7K8M, 7K90, 7JX3, 7JN5, 6ZER). *nAb not described in this paper.

The epitopes in the RBD are divided into different classes based on their binding region. A number of different classification systems have been proposed, but the two most commonly referenced are those proposed by Piccoli et al. (1) and Barnes et al. (5, 18). Piccoli et al. (1) proposed 6 epitopes termed Ia, Ib, IIa, IIb, IIc and IV, while Barnes et al. (5, 18) grouped the antibodies into 4 classes based on their binding mode to the Spike protein (**Figure 3**). There are some overlaps between these 2 classifications in which epitope 1a corresponds to class 1 antibody binding site, epitope 1b to class 2 antibody, epitope IV to Class 3, epitope IIb and IIc roughly lie on the Class 4 binding site (1, 5, 18–20). Class 1 antibodies are commonly derived from VH3-53/VH3-66 germlines, contain a short CDR-H3 and compete with ACE2 for binding site and only recognize ‘up’ RBD (**Figure 1**). Class 2 has a long CDR-H3 loop and are capable of binding to RBD in both ‘up’ and ‘down’ conformations. As with Class 1, they also compete with ACE2 for RBD binding. Class 3 Ab bind to the RBD on the opposite side of Class 1 and Class 2 binding epitope close to an N-glycan attached to residue N343. Class 4 antibody, usually with poor neutralizing activity, target a cryptic epitope that faces the interior of the S protein on ‘up’ RBDs (5, 18). Some Class 4 Abs have recently been reported to potently neutralize a broad spectrum of β-coronaviruses including RaTG13, Pangolin Guangdong, SARS-CoV 1, SARS-CoV 2, WIV1 and SHC014 and importantly appear to retain their neutralizing function against the novel SARS-CoV-2 mutants (21, 22).

### SARS-COV-2 Evolution And Impact On Recognition by Neutralizing Antibodies

The emergence of mutant SARS-CoV-2 variants and their impact on the efficacy of nAbs and vaccines has become a major concern in the current progress of the pandemic (23–25). Immune selection pressure during protracted infections are presumed to have contributed to the emergence of these variants (26). Although as the levels of herd immunity increase through natural and vaccine induced immunity it is reasonable to speculate that this will provide greater selection pressure on the virus and we may see more mutants continue to emerge. Due to their potential to transform the strength and kinetics of the binding with ACE2, mutations that arise in the RBD are of particular interest. The earliest variant to emerge and rapidly became dominant worldwide carried a D614G substitution and although studies suggested increased transmissibility (27–29), this variant was neutralized with existing monoclonal antibodies and convalescent sera (30–32). The emergence of the N501Y substitution was particularly concerning, which has been reported to be more transmissible than wild type and the D614G substitution (11, 33, 34). The N501Y substitution was first seen in the B.1.1.7 variant (23) and subsequently in the B.1351 (24) and P1 variants (35). The other concerning immune escape substitution in the RBD is E484K, which is also present in the B.1.351 and P.1 variants. The B.1351 and P.1 variants also harbor K417N and K417T substitution, respectively (23–24, 35). The presence of the N501Y substitution has been reported to increase affinity to ACE2 7-fold, and the additive combination of substitutions at 417, 484, and 501 have shown further increased affinity to ACE2 (19-fold compared to Wuhan) (11, 36). The other significant RBD substitutions are Y453F in the B.1.1.298 variant (37), L452 in the B.1.429 (25) and B.1.617 variants (38). Recently, WHO has renamed the variants by using Greek letters to refer to the variants as: B.1.1.7 as Alpha, B.1.351 as Beta, P1 as Gamma and B.1.617.2 as Delta (39, 40) (**Table 1**). A variant is characterized as a variant of concern (VOC) if it demonstrates increased transmissibility, increased virulence, change in disease presentation, or reduced effectiveness of: vaccines, diagnostic testing and treatment measures (40).

**Table 1:** WHO designation of variants (40).

WHO label	Pango lineage	Current status
Alpha	B.1.1.7	Variants of concern
Beta	B.1.351	Variants of concern
Gamma	P1	Variants of concern
Delta	B.1.617.2	Variants of concern
Eta	B.1.525	Variants of interest
Iota	B.1.526	Variants of interest
Kappa	B1.617.1	Variants of interest
Lambda	C.37	Variants of interest
Mu	B.1.621	Variants of interest

These circulating VOCs of SARS-CoV-2, including B.1.1.7 (23), B.1.351 and P.1 (35, 41) show decreased susceptibility to some SARS-CoV-2 mAbs (11, 42–44), convalescent plasma (11, 45) and sera from SARS-CoV-2 vaccinees (11, 36, 44, 46, 47). The Delta variant, B.1.617.2, has become the dominant SARS-COV-2 variant worldwide (48) and is associated with increased viral replication leading to increased transmissibility, higher viral load and severity (49, 50). The emergence of B.1.617.2 is associated with evasion of mAbs and vaccine efficacy (50, 51). The availability of therapeutic nAbs effective against all SARS-CoV-2 variants will offer benefits for the control of the current pandemic variants and future variants, and their development therefore remains a high priority (52). The presence of broad mAbs have been described in convalescent patients and offer hope that the current vaccines might be effective (53). Most of the mAbs isolated from convalescent patients are Class 1 antibodies (18, 19). The majority of broadly-neutralizing antibodies (bnAbs) described to-date also fall within this same class (9–11), however, bnAbs belonging to Class 4 with high potency and neutralizing breadth have been characterized (21, 22). Class 2 comprises of some potent Abs (C144, C121, COVA2-15, COVA2-37), however, their efficacy against emerging variants have not been described (19, 54). Class 3 antibodies bind to a conserved epitope and are generally unaffected by mutations (19, 55). Understanding the different classes of broad mAbs that are generated and their persistence in convalescent recovered patients and vaccines is important for understanding the robustness of the herd immunity that is generated. Here we review some of the most promising broadly neutralizing antibodies and their mode of interaction obtained from convalescent patients. It will be important to understand how many of these broad neutralizing antibodies are retained post vaccination and natural infection.

## Classification of Antibodies Based on Their Binding Mode to the RBD of the Spike Protein

In this review, we will primarily use the Barnes classification, although the classifications by Piccoli et al. will be occasionally cross-referenced (**Table 2**).

**Table 2:** Monoclonal antibodies, their classification and mechanism of action.

mAbs	VH-class	CHR-H3 sequence	Binding site	Epitope (Piccoli, Barnes)	Residues	Mechanism of action	Variants	Live virus IC50 (ng/ml)	Pseudovirus IC50 (ng/ml)	Reference
COVOX-222	VH3-53	ARGEGSPGNWFDP	RBD	Ia, Class 1	403,417, 420-21, 455,457-58, 473, 475, 477, 487, 502, 505	Inhibition of RBD ACE2 interaction	B.1.1.7, B.1.351, P1	WT: 16		(10)
COVOX-253	VH1-58	AAPHCNSTSCYDAFDI	RBD	1a, Class 1	458, 473, 475, 477-78, 486-87	Inhibition of RBD ACE2 interaction	B.1.1.7, B.1.351, P1	40		(9, 11)
A23-58.1	VH1-58	AAPNCSNVVCYDGFDI	RBD	1a, Class 1	475, 477, 478, 487	Inhibition of RBD ACE2 interaction	B.1.1.7, B.1.351, P1	2.1	2.5	(56)
							B.1.617.1,			
							B.1.617.2, B.1.427, B.1.429			
B1-182.1	VH1-58	AAPYCSGGSCFDGFDI	RBD	1a, Class 1	475, 477, 478, 487	Inhibition of RBD ACE2 interaction	B.1.1.7, B.1.351, P1	2.4	3.4	(56)
							B.1.617.1,			
							B.1.617.2, B.1.427, B.1.429			
Fab 1-57	VH3-72	ARVHRWAYCINGVCFGAYSDY	RBD	Ia, Class 1	446-47, 449-50, 470, 484, 493, 498	Inhibition of ACE2 binding to RBD	B.1.1.7	8		(57)
							B.1351			
Fab 2-7	VH2-5	AHHKIERIFDY	RBD	1a, Class I	440, 444-47, 450, 500	Inhibition of ACE2 binding to RBD	B.1.1.7	3		(57)
							B.1351			
BG10-19	VH5-59	ARTQWGYNYGSHFFYMDV	RBD	1a, Class I	346, 373, 437, 440-41, 444, 448,	Locks the S timer into closed confirmation thus masking the RBD	B.1.1.7	–	WT: 0.002	(58)
							B.1.351		B.1.1.7:0.001	
									B.1.351: 0.004	
54042-4	VH2-5	AHGLFSSSDWGGLDV	RBD	Ia, Class 1	441, 443-47, 498-500	Inhibits interaction of RBD to ACE2	B.1.17, B.1.351, P1, B.1.141, B.1.258	WT: 3.2	WT:9	(59)
								B.1.1.7: 7.2		
								B.1.351: 13		
LY-COV555	VH1-69	ARGYYEARHYYYYYAMDV	RBD	Ib, Class 2	482, 484, 485, 486, 489, 493, 494	Inhibits interaction of RBD to ACE2	B.1.1.7,		WT: 0.012	(60)
							B.1.351, P1			
MD65	VH3-66	ARDLAVAGAFDI	RBD	Ib, Class 2	452, 484, 486, 490, 493, 494	Inhibits interaction of RBD to ACE2	B.1.1.7, B.1.351		WT: 0.1	(61)
									B.1.1.7: 0.04	
									B.1.351: 0.4	
LY-CoV1404	VH2-5	AHHSISTIFDH	RBD	IV, Class 3	346, 439-40, 444-45, 447, 450, 498, 500	Inhibits interaction of RBD to ACE2	B.1.1.7	9-22	WT: 3	(62)
							B.1.351		B.1.1.7: 2	
							P1		B.1.351:9	
							B.1.427		P1: 5	
							B.1.526		B.1.427: 8	
									B.1.526: 19	
S2X259	VH1-69	ARGFNGNYYGWGDDDAFDI	RBD	II, Class 4	370, 374, 377-79, 383, 385, 405, 408, 503, 504	Inhibition of ACE2 binding to RBD	B.1.1.7, B.1.351, P1, B.1.427, B.1.429	144.2	WT: 213.2	(22)
									B.1.1.7: 205	
									B.1.351: 357	
									B.1.429: 114	
									P1: 458	
C118	VH3-30	ASGYTGYDYFVRGDYYGLDV	RBD cryptic epitope	IIb, Class 4	355-56, 364-66, 391-95, 399-402, 414-17	Compete with ACE2 for RBD binding	B.1.17, B.1.351, B.1.429, B.1.526	<1		(21)
C022	VH4-39	ARHAAAYYDRSGYYFIEYFQH	RBD, cryptic epitope	IIb, Class 4	368, 374-79, 408-9, 412-17, 427-29, 446	Compete with ACE2 for RBD binding	B.1.17, B.1.351, B.1.429, B.1.526	<1		(21)
S2P6	VH1-46	ARGSPKGAFDY	Stem helix of S2 subunit	NA	1146-1159	Disrupts stem helix bundle, inhibits membrane fusion	B.1.1.7, B.1.351, P1		WT: 1.4	(63)

WT, wild type (SARS-COV-2 Wuhan strain); RBD, receptor binding domain

### Class 1 Antibodies

Class 1 Abs are the most immunodominant among RBD-targeting antibodies and are usually encoded by VH3-53 and VH3-66 germlines (5, 10, 17, 54). The family of IGHV3-53 antibodies have been described to share common binding properties and bind to a common epitope around the neck of the RBD, with an identical approach also shared by the IGHV3-66 derived Abs (5, 11, 36). The binding and neutralization of this Class of antibodies are usually abrogated by N501Y, E484K and K417N mutations (64). Their engagement with the RBD is dictated by CDR-H1 and CDR-H2, while the CDR-H3 is short and makes few interactions (5, 10, 65). However, CDR-H3 does interact with K417 and CDR-L1 with N501, and therefore, the neutralization activity of many class I VH3–53 antibodies are compromised by the N501Y substitution in the variant viruses B.1.1.7, B.1.351, and P.1, while the added substitution at 417 in P.1 and B.1.351 has an additive negative effect on neutralizing activity (10). Hence, the neutralizing potency of the majority of the Class I mAbs are markedly reduced against the variants (66). Class 1, however, also contains several nAbs capable of neutralizing the emerging variants. In this section we discuss several of these antibodies and describe their interaction with the SARS-CoV-2 Spike.

Dejnirattisai et al. isolated the class I mAb, COVOX-222, which is derived from VH3-53, that neutralizes all three variants despite binding with two of the ACE2 binding site substitutions (10, 36) (**Table 2**). In the original virus, residue 417 makes a weak salt-bridge interaction with heavy-chain CDR3 residue E99 which is abolished due to a substitution to either asparagine or threonine (10). The CDR-H3 of COVOX-222 (13 residues) is slightly longer than the majority of VH3–53 antibodies; however, this seems unlikely to be the only reason for the resilience of COVOX-222 (10, 65). There is little binding energy from the CDR3-H3, since majority of the binding energy input of the heavy chain comes from CDR-H1 and CDR-H2, which interacts weakly with RBD residue 417. Therefore many of the VH3–53 antibodies are likely to be volatile to mutations at residue 417 (K417N in B.1.351 and K417T in P1). The COVOX-22 CDR L1 interacts with residue 501 of the RBD through its P30 residue. The interaction is further strengthened by the N501Y substitution, that eventually adds to the resilience of this antibody (10). Hence, COVOX-222, a mAb of Class 1, VH3-53 gene family, despite its short CDR-H3 and binding with mutant RBD residues, is able to neutralize SARS-COV-2 variants.

Another group of class I mAbs, referred to as 55, 165, 253 and 318, have also been reported to retain nAb activity against the variants (67). These four mAbs are all IGHV1-58 class, have fewer non-silent mutations (2–5) and a longer heavy chain CDR3 (12–16 residues). mAb 55, 165 and 253 bind to the dominant neck epitope of the RBD while 318 binds to an epitope at the right shoulder. The FRNT_50_ titers for mAbs 55, 165, 253, and 318 are also relatively equal between Victoria strain (A.1) (67) and B.1.351, indicating that their epitopes are not impacted by the K417N, E484K and N501Y mutations (9, 11). These mAbs, despite different VH gene usage as compared to the COVOX-222, are still able to neutralize the wild type SARS-COV-2 and existing variants.

A separate group of researchers isolated two antibodies, A23-51.1 and B1-182.1, from convalescent subjects infected with the Washington-1 (WA-1) strain, which has an identical S sequence to Hu-1 (56). These antibodies showed the capacity to maintain high neutralization potency against 10 variant spike proteins including B.1.1.7, B.1.351, P1 and the highly transmissible B.1.617.2 variant. The two antibodies, A23-58.1 and B1-182.1, share similar gene family usage in their heavy and light chains; both use IGHV1-58 heavy chains and IGKV3-20/IGKJ1 light chains and low levels of SHM. Both of these mAbs have a similar mode of binding to RBD. While binding, the RBD residue 486 dip into the crater formed by the CDRs and form a hook-like motif that is stabilized by an intra-loop disulfide bond between residues 480 and 488 while aromatic residues, including 456, 473, 486 and 489 provide 48% (299 Å^2^) of the epitope. In comparison to epitopes of other antibodies, the supersite defined by common contacts of these IGHV1-58-derived antibodies had fewer interactions with residues at the mutational hotspots. The hook-like motif and CDR crater are essential for the binding within the VH1-58 public class (56). This antibody gene family combination has been proposed to be a public clonotype as it has been identified in other COVID-19 convalescent subjects (6, 9, 54).

Another study recently characterized six potently neutralizing antibodies from B cells from convalescent subjects (58). Among them, BG10-19 potently neutralized the wild-type SARS-CoV-2, B.1.1.7 (23), B.1.351 as well as the heterologous SARS-CoV pseudotyped viruses (58). BG10-19 uses five of six CDR loops to connect with a proteoglycan epitope directed up on the RBD α-1 (338–347) and α-2 (364–374) helices, with additional contacts with 436-450. The CDRH2 and CDRH3 loops moderate the majority of RBD contacts (~760Å2), establishing strong interactions with RBD residues. CDRH1-3 and CDRL2 loops interaction establish the primary epitope recognized by BG10-19, which does not overlap with the ACE2 receptor-binding motif. Overall, BG10-19 employ a neutralization mechanism that masks engagement of the ACE2 by RBM, by locking the Spike trimer into a closed conformation (58).

Another Class I mAbs of interest are monoclonal antibodies Fab 1-57 and Fab 2-7 which bind to the RBD epitope outside the hotspot of evolutionary pressure (57). Recognition of wild-type RBD by Fab 1-57 is dominated by the heavy chain, which buries 533.7 Å surface area, with a minor 223.3 Å contribution by the light chain. With respect to the mutations in the B.1.1.7, B.1.351 and P1 variants, only residue 484 was near the binding site of Fab1-57. Despite its proximity to the epitope, however, 484 do not interact significantly with Fab 1-57. Structural modeling of the E484K mutation showed that the K484 residue was geometrically compatible with Fab 1-57 binding at serine 29 with a hydrogen bond (57). Interaction of wild-type RBD by antibody Fab 2-7 is dominated by connection proximal to the RBD loops formed by residues 438-451 and 495-502. CDR H2 residues 54, 52, and 58 formed hydrogen bonds with RBD residues 450, the backbone amine of 445, and 447, respectively. Light chain residue 32 also interacted with residue 440 to form a hydrogen bond. Regarding the three mutated residues, antibody Fab 2-7 bound near only N501, but the side chain of N501 pointed away from the antibody. Even though some conformational change of the 495-502 loop would occur due to N501Y mutation, this loop contributes only 225 Å2 out of 736 Å2 and contained few residues that form significant interactions with the Fab (57). The therapeutic use of Abs, such as Fab 1–57 and 2–7, which target less prevalent epitopes, could mitigate concern of mAb escape.

Along with the existing methods, newer technologies are also being deployed for this purpose (68). Kramer et al. (59) used LIBRA-seq technology (69) and identified a potent monoclonal antibody 54042-4 from a convalescent COVID-19 patient that bound and neutralized the live SARS-CoV-2 viruses, including variants of concern. They concluded that antibody 54042-4 bound to these RBD variants (B.1.17, B.1.351, P1, B.1.141, B.1.258) at a level comparable to the binding to the RBD of Wuhan-1 isolate. The cryo-EM structure showed that 54042-4 forms a vast interface with the RBD through all three CDRs of the heavy chain, CDRL1 and CDRL3 to form a clamp over the apex of the RBM saddle. The heavy chain forms interaction with RBD residues 443-447, while light chain interacts with residues 445 and 498-500. CDR H1 binds to 441, CDR H2 to 444 and CDR H3 to 443. CDR L1 and L3 makes hydrogen bond with 445 and 498-500. The complex interaction indicated that the spike substitutions in current VOCs are unlikely to affect the binding affinity of 54042-4. RBD residue N501 lies outside of the 54042-4 epitope, while the Cα atoms of E484 and K452 are 18 and 14 Å far from the nearest 54042-4 residue, respectively. The use of modern technology like LIBRA-seq enables high-throughput concurrent determination of B cell receptor sequence and antigen reactivity at the single-cell level, facilitating the candidate selection and characterization process, and also expedite the development of broadly-neutralizing antibodies.

Class 1 is comprised of a diverse array of mAbs with strong neutralization breadth and potency. Despite variation in their VH gene usage, all the above mAbs can effectively neutralize the emerging variants of SARS-COV-2. These antibodies could be used alone or in combination with other classes of antibodies in manufacturing therapeutic antibodies and also guide the formulation of next generation vaccines.

### Class 2 Antibodies

Class 2 comprises of some potent Abs (C144, C121, COVA2-15, COVA2-37) that can bind to the RBD in both the up and down confirmation. In general, through their efficacy against emerging variants have not been described (19, 54). However, two Class 2 mAbs and the impact of known mutations have been well described. LY-CoV555 (clinical name bamlanivimab), currently used in the clinical setting in combination with etesivimab, is a potent anti-spike neutralizing antibody (70, 71). Structural analysis revealed that it binds both in up and down confirmation of the RBD, and the binding of this mAb to RBD is not affected by the N501Y mutation; however, the E484K mutation abolishes binding (60). Against B.1.351, activities of bamlanivimab and another therapeutic mAb REGN10933 belonging to Class 1 antibodies are also abolished (66). Another Class 2 antibody, MD65, has a binding pattern similar to LY-CoV555 but unlike LY-CoV555, the E484K mutation did not affect its binding efficacy (61). The *in vivo* assessment on K18-hACE2 transgenic mice suggested that MD65 exhibited efficacy against B.1.1.7, B.1.351 and P1 variant (61). So, it appears that broadly reactive Class 2 mAbs can be developed despite the shortcomings of bamlanivimab against B.1.351.

### Class 3 Antibodies

Antibodies targeting the Class 3 epitope can bind with the RBD in both “up” and “down” states (18, 19). The class 3 epitope is highly conserved in Sarbecovirus clades 1, 2, and 3, indicating it is a good target for broad neutralizing antibodies and suggests it is functional conserved and less likely to be associated with immune (55, 72). The Class 3 antibodies bind outside of the ACE-2 binding region and hence, provide the potential for synergistic effects when combined with nAbs that intercept ACE2 binding.

The Class 3 monoclonal antibody LY-CoV1404 was isolated from a high-throughput screen of peripheral blood mononuclear cells obtained from a convalescent subject 60 days after symptom onset (62). Using authentic and pseudoviruses neutralization assays, researchers showed that LY-CoV1404 maintains potent neutralizing activity against multiple variants including B.1.1.7, B.1.351, B.1.427, P.1 and B.1.526. Interestingly, LY-CoV1404 share 92% amino acid sequence identity in the variable regions of both its heavy and light chains, to antibody Fab 2-7 (57), though they were discovered independently from different patients suggesting it maybe a publicly shared repertoire (62). Both antibodies also append to the RBD in a similar way. LY-CoV1404 binds to a region overlapping the ACE2-interacting site of the spike that is accessible in open and closed state of the RBD. While this property would advise that this is a Class 2 antibody (5, 18), the location of the epitope is identical to mAb S309, a class 3 binder (55). Although LY-CoV1404 binding epitope includes residues N501 and N439, they can bind the B.1.1.7 and B.1.351 variants and are neutralized as strongly as wild type virus (62). The above findings suggest that LY-CoV1404 represents a potent mAb with broad neutralizing range, possess the property of both Class 2 and 3 antibodies, has a relatively conserved epitope and could well be deployed to address the concern of emerging variants. LY-CoV1404’s potent neutralization of SARS-CoV-2 allows for exploration of lower clinical doses, which may support subcutaneous administration and has the potential to provide a long-term complement to vaccines in the likely event that COVID-19 becomes endemic. Class 3 nAbs supplement the anti-SARS-CoV-2 antibody repertoire and can be effectively utilized in therapeutic combinations with class 1 or 2 nAbs (18).

Another group of antibodies in class 3 are VIR-7831 and VIR-7832; dual action mAbs derived from the parent antibody S309–an antibody obtained from a SARS-CoV survivor (55). These mAbs have been engineered to have an extended half-life and improved lung bioavailability (73) and target to an epitope located around residue N343 that is highly conserved among the Sarbecovirus, and neutralize live and pseudotyped virus against B.1.1.7, B.1351 and P1 variants. They also exhibit potent effector function and confer antibody dependent cellular cytotoxicity and antibody dependent cellular phagocytosis *in vitro* (74). The mAb VIR-7831 has been provided emergency use authorizations (EUA) by the US FDA for treatment of mild to moderate COVID-19 (75).

### Class 4 Antibodies

Class 4 Abs bind to the highly conserved, cryptic epitope on the RBD outside the RBM (18). The majority of mAbs described previously are cross-reactive but weakly neutralizing (1, 17, 76–78). However, Jette et al. (21) characterized two Class 4 anti-RBD antibodies, C118 and C022, that were obtained from COVID-19 donors and revealed broad recognition and potent neutralization of SARS-CoV-2 variants (21). They found that C118 and C022 Abs neutralized four SARS-CoV-2 variants (B.1.17, B.1.351, B.1.429, B.1.526). The structure analysis showed that both mAbs recognized an epitope that is highly conserved at the base of the RBD, which is disclosed only in ‘up’ conformations. C118 and C022 use four of six CDR loops to bind to the epitope that stretches towards the RBD ridge close to the ACE2 binding region, and C022 includes an additional overlapping interacting residue at 417. CDRH3 and CDRL2 loops, and portions of framework region L3 of both antibodies dominate the RBD contacts and develop polar and van der Waals interactions that accounts for 71% of the epitope buried surface area (21). C022 and C118 form considerable backbone interactions with RBD, with 9 and 10 H-bonds formed with the RBD, respectively contributing to their cross-neutralization across variants and breadth, as these interactions would mediate binding despite side chain substitutions.

The isolation and characterization of nAbs targeting conserved RBD epitopes that possess dual advantage of breadth across Sarbecoviruses and higher resistance to neutralization escape is necessary to fight this pandemic (79). Due to the conservation at the Class IV epitope, antibodies targeting this site are of interest. S2X259, is another class 3 antibody isolated (22) from a convalescent patient which broadly neutralizes entry of SARS-CoV-2 including the B.1.1.7, B.1.351, P.1 and B.1.427/B.1.429 variants. This antibody acts through inhibition of ACE2 binding to the RBD. They also performed several experiments to show that this antibody is effective against a wide spectrum of human and zoonotic sarbecoviruses and retain a high barrier to the emergence of resistant mutants (22). S2X259 targets a glycan-free, cryptic epitope within antigenic site IIa and binds with the RBD using both heavy and light chains contributing two thirds and one third of the paratope surface buried upon binding, respectively (1, 22). S2X259 uses CDR H1-H3, L1 and L3 to interact with residues 369-386 forming two α-helices and an intervening β-sheet. The S2X259 epitope is highly conserved in SARS-CoV-2 viruses and does not contain prevalent mutant residues, such as L452R, S477N or N439K. The mAb binds with the backbone of residue N501 and not its side chain, circumvents the residues 417 and 484 which could explain the preserved neutralizing potency against B.1.1.7, P1, B.1.351 and B.1.429 (22).

Due to its conserved and cryptic epitope, Class 4 antibodies are better assets for neutralization across multiple variants and thereby potentially protect against emergent Sarbecoviruses (21, 22).

## Monoclonal Antibody Binding To Stem Helix of S2 Subunit

Abs binding to the RBD and NTD within the S1 subunit wield a selective pressure resulting in the emergence of variants. Due to the low pressure and necessity to sustain the functionality of fusion machinery, the S2 subunit is highly conserved. Pinto et al. isolated five mAbs from three convalescent COVID-19 patients that were broadly neutralizing against the SARS-COV-2, mutants and other closely related viruses, the most potent being mAb S2P6 (63). These mAbs targeted the stem helix within the S2 subunit of the Spike protein and cross-reacted with human and animal β-coronaviruses. They assessed S2P6-mediated neutralization of SARS-CoV-2 wild-type and variants B.1.1.7, B.1.351 and P.1, and found identical potency to that found against the D614G variant (63). The stem helix peptide folds as an amphipathic α-helix resolved for residues 1146 to 1159. Upon binding to the epitope, S2P6 buries approximately 600 A^2^ involving CDR H1-H3, L1 and L3. The hydrophobic side of the stem helix docks with the mAb S2P6 *via* residues 1148, 1152, 1155 and 1156 with residues 33, 93, 96 and 99 of heavy chain and residues 50, 57-59, 101-103 of light chain (63). Structural and functional experiments concluded that S2P6 disrupts the stem helix bundle and prevents viral entry *via* inhibition of membrane fusion resulting from impeding S fusogenic rearrangements (63). The exceptional neutralization breadth and cross-reactivity of S2P6 is due to the conserved nature of the stem helix among β-coronaviruses. Owing to its low selection pressure and conserved epitope far from the binding site of ACE2 and other mAbs, these antibodies targeting the S2 subunit could well offer an additional therapeutic option.

### NTD-Directed Monoclonal Antibodies

The US FDA has issued EUA for several mAb therapies for the management of COVID-19 and these are solely targeting the RBD. However, the widespread circulation of variants has led to the withdrawal of EUAs for some mAb monotherapies accentuating the demand to expand combination therapies that can counter emerging variants and curtail the mutational escape (80, 81). Of late, some studies have reported a few NTD targeting mAbs and demonstrated their ability to mitigate SARS-CoV-2 infection (12, 16), although their neutralization profile is generally less potent. It is unclear how the NTD-directed mAbs inhibit infection (16, 82); however, it has been postulated that the extensive N-linked glycan shielding (83) and inability to compete with ACE2 makes them poor neutralizers as compared to RBD directed mAbs (16). Several SARS-CoV-2 variants, including B.1.1.7, B.1.351, and P1, harbor mutations within the NTD supersite which may limit the potential for broad nAb recognition at this site (13, 82, 84). Some promising NTD-directed antibodies like 4A8 (12), S2M28 (82), S2X28 (82), BLN14 (61), COV2-2676, COV2-2489 (85) and ADI-56479 (80) have been described recently; however, their potency against the VOCs, when used as monotherapy, are not well characterized. The researchers suggest that these mAbs could be used in cocktail therapies with RBD-directed antibodies to raise the barrier to immune escape (12, 80, 85).

## Role of Memory B Cell

It is apparent that bnAbs can be generated against several different epitopes of SARS-CoV-2. However, it is not yet clear how prevalent bnAbs are across the population and how well they are maintained in the memory B cell pool. Recently, Nussenzweig et al. (53) conducted a longitudinal study in 63 COVID-19 convalescent patients up to 1 year and revealed that the neutralizing potency and number of RBD-specific memory B cells remained stable up to 1 year. Importantly, anti-RBD B cells cross-reactive to the VOCs were present but were 1.6 to 3.2-fold lower than wild-type RBD binding B cells. Among patients who received mRNA vaccines, however, memory B cells increased 8.6-fold. They concluded that immunity in convalescent individuals will be long lasting and that convalescent individuals receiving mRNA vaccines will produce antibodies and memory B cells protective against circulating SARS-CoV-2 variants (53). These findings were supported by another study by Winkmeier et al., who assessed cross-variant memory-B-cell responses among 16 convalescent patients and found that memory-B-cell-derived IgGs recognized the RBD of B.1.1.7 similarly to the wild-type, while reactivity to B.1.351 and P.1. decreased by 30% and 50%, respectively (86). Taken together, despite declining antibody titers, memory B cells with broad cross-reactivity have been shown to persist up to 1 year after natural infection and are capable of producing nAbs against wild type and variant RBDs. While memory B cells may not protect against breakthrough infection, antibodies produced by these memory B cells might ameliorate severe disease. It will be important to determine the prevalence of bnAbs in the long lived memory B cell pool to understand the breadth of protection.

## Conclusion

SARS-CoV-2 variants escape neutralization by antibodies from vaccine and natural infection, which highlight the urgent need for a wide range of potently neutralizing antibodies against variants (42, 44, 47, 87). The ability of SARS-CoV-2 variants to negatively alter the trajectory of the pandemic highlight the urgent need for antibody therapies that can be developed in real time to counter the virus as it evolves. The rapid evolution of SARS-COV-2 escape mutants have resulted in reduced efficacy of most available vaccines and monoclonal antibodies. In this review, we have discussed several monoclonal antibodies characterized from convalescent patients which are potent neutralizers and resistant to mutations. These bnAbs could be used as therapeutic options for the treatment of severe COVID-19 patients and can effectively complement the vaccines in the containment of current pandemic. These passively administered mAb can act in combination with the host immune response to evade the development of severe COVID-19 and limit onward transmission.

## Author Contributions

Concept, LS. Writing, LS and RB. Supervision and review, RB and NT. All authors contributed to the article and approved the submitted version.

## Funding

RB is funded by NHMRC investigator grant (Grant ID: GNT1195720).

## Conflict of Interest

The authors declare that the research was conducted in the absence of any commercial or financial relationships that could be construed as a potential conflict of interest.

## Publisher’s Note

All claims expressed in this article are solely those of the authors and do not necessarily represent those of their affiliated organizations, or those of the publisher, the editors and the reviewers. Any product that may be evaluated in this article, or claim that may be made by its manufacturer, is not guaranteed or endorsed by the publisher.
